# Micro-CT-Based Quantification of Extracted Thrombus Burden Characteristics and Association With Angiographic Outcomes in Patients With ST-Elevation Myocardial Infarction: The QUEST-STEMI Study

**DOI:** 10.3389/fcvm.2021.646064

**Published:** 2021-04-21

**Authors:** Efstratios Karagiannidis, Andreas S Papazoglou, Georgios Sofidis, Evangelia Chatzinikolaou, Kleoniki Keklikoglou, Eleftherios Panteris, Anastasios Kartas, Nikolaos Stalikas, Thomas Zegkos, Fotios Girtovitis, Dimitrios V. Moysidis, Leandros Stefanopoulos, Kleanthis Koupidis, Stavros Hadjimiltiades, George Giannakoulas, Christos Arvanitidis, James S. Michaelson, Haralambos Karvounis, Georgios Sianos

**Affiliations:** ^1^First Department of Cardiology, AHEPA University Hospital, Aristotle University of Thessaloniki, Thessaloniki, Greece; ^2^Hellenic Centre for Marine Research, Institute of Marine Biology, Biotechnology, and Aquaculture, Heraklion, Greece; ^3^Biology Department, University of Crete, Heraklion, Greece; ^4^Laboratory of Forensic Medicine and Toxicology, School of Medicine, Aristotle University of Thessaloniki, Thessaloniki, Greece; ^5^Blood Centre, AHEPA University Hospital, Thessaloniki, Greece; ^6^Lab of Computing, Medical Informatics, and Biomedical Imaging Technologies, Aristotle University of Thessaloniki, Thessaloniki, Greece; ^7^School of Mathematics, Aristotle University of Thessaloniki, Thessaloniki, Greece; ^8^LifeWatch ERIC, Sector II-II, Seville, Spain; ^9^Massachusetts General Hospital and Harvard Medical School, Boston, MA, United States

**Keywords:** micro-computed tomography, thrombus aspiration, thrombus, ST-elevation myocardial infarction, interventional cardiology

## Abstract

**Background:** Angiographic detection of thrombus in STEMI is associated with adverse outcomes. However, routine thrombus aspiration failed to demonstrate the anticipated benefit. Hence, management of high coronary thrombus burden remains challenging. We sought to assess for the first time extracted thrombotic material characteristics utilizing micro-computed tomography (micro-CT).

**Methods:** One hundred thirteen STEMI patients undergoing thrombus aspiration were enrolled. Micro-CT was undertaken to quantify retrieved thrombus volume, surface, and density. Correlation of these indices with angiographic and electrocardiographic outcomes was performed.

**Results:** Mean aspirated thrombus volume, surface, and density (±standard deviation) were 15.71 ± 20.10 mm^3^, 302.89 ± 692.54 mm^2^, and 3139.04 ± 901.88 Hounsfield units, respectively. Aspirated volume and surface were significantly higher (*p* < 0.001) in patients with higher angiographic thrombus burden. After multivariable analysis, independent predictors for thrombus volume were reference vessel diameter (RVD) (*p* = 0.011), right coronary artery (RCA) (*p* = 0.039), and smoking (*p* = 0.027), whereas RVD (*p* = 0.018) and RCA (*p* = 0.019) were predictive for thrombus surface. Thrombus volume and surface were independently associated with distal embolization (*p* = 0.007 and *p* = 0.028, respectively), no-reflow phenomenon (*p* = 0.002 and *p* = 0.006, respectively), and angiographically evident residual thrombus (*p* = 0.007 and *p* = 0.002, respectively). Higher thrombus density was correlated with worse pre-procedural TIMI flow (*p* < 0.001). Patients with higher aspirated volume and surface developed less ST resolution (*p* = 0.042 and *p* = 0.023, respectively).

**Conclusions:** Angiographic outcomes linked with worse prognosis were more frequent among patients with larger extracted thrombus. Despite retrieving larger thrombus load in these patients, current thrombectomy devices fail to deal with thrombotic material adequately. Further studies of novel thrombus aspiration technologies are warranted to improve patient outcomes.

**Clinical Trial Registration:** QUEST-STEMI trial ClinicalTrials.gov number: NCT03429608 Date of registration: February 12, 2018. The study was prospectively registered.

## Introduction

Despite the tremendous progress in cardiovascular medicine over the last decades, ST-segment elevation myocardial infarction (STEMI) still remains one of the leading causes of mortality worldwide (1). Intracoronary thrombosis developed after plaque erosion or rupture, causing partial or total occlusion of coronary vessels which is the most common underlying pathophysiologic mechanism in STEMI. Thrombus burden (TB) is an important prognostic determinant (2), as it has been associated with an increase in the rate of major adverse cardiac and cerebrovascular events (MACCE) (3).

The optimal therapy for STEMI is timely performed primary percutaneous coronary intervention (pPCI) (4). However, myocardial recovery and restoration of epicardial coronary blood flow are often suboptimal due to thrombus embolization (5), leading to perturbed microvascular perfusion and obstruction of the microvasculature.

Manual aspiration thrombectomy (MATh) was first described in 1980 as a useful adjunctive therapy to conventional PCI with the potential to remove the thrombotic component of the culprit lesion (6, 7). However, large randomized controlled trials (RCTs) and meta-analyses failed to demonstrate the theoretically anticipated benefit for routine MATh, suggesting lack of synergy between MATh and pPCI with a subsequent increase in the risk of stroke (8–11). Therefore, current ESC guidelines do not recommend routine thrombus aspiration (class of recommendation IIIA) (12).

Several hypotheses might serve to explain why MATh did not succeed in the majority of recent trials, such as the presence of small amount of thrombus at the culprit lesion before thrombectomy or of large amount of residual thrombus after thrombectomy (13). Indeed, it was shown that the elective application of MATh in certain cases with large thrombus burden (9, 14, 15) as a bail-out therapy showed some cardiovascular (CV) benefit (reduced CV death), which was counterbalanced by an increased risk of stroke. Previous studies utilizing optical coherence tomography (OCT) have shown that there is substantial amount of residual thrombus even after thrombectomy (13, 14, 16). Interestingly, STEMI patients with greater residual TB after MATh had microvascular dysfunction and more significant myocardial damage than those with smaller residual TB (14).

Thus, the prognostic significance of initial angiographic TB and post-aspiration residual TB has already been investigated. However, evidence on the association of extracted thrombotic material characteristics with post-pPCI angiographic outcomes is lacking.

Micro-computed tomography (micro-CT) is an emerging technology with high spatial resolution in the submicrometer range, which is increasingly employed in medicinal studies (17–20). Despite being initially used for skeletal imaging, the development of contrast agents, which amplify the low intrinsic contrast of soft-tissues in X-ray absorption, facilitates detailed micro-CT imaging of soft tissues (21). Since micro-CT allows non-destructive 3D imaging of both the internal and external structures of samples, exceeding the capabilities of histomorphometric analysis (21), it can be employed to accurately quantify extracted thrombotic material characteristics, which have been subjective to date. These novel imaging parameters could be used to improve patient risk stratification, enabling individualized treatment of patients with STEMI.

## Materials and Methods

The design of the QUEST-STEMI study has been previously described (22).

### Study Population and PCI Procedures

QUEST-STEMI (ClinicalTrials.gov Identifier: NCT03429608) is a prospective cohort trial including patients, who presented to AHEPA University Hospital with STEMI and underwent primary PCI and MATh at the discretion of the treating physician within 12 h of symptom onset. The eligibility criteria are depicted in Table 1. The Scientific Committee of AHEPA Hospital approved the study protocol, and all trial procedures comply with the principles set by the Declaration of Helsinki (23). Each participant provided written informed consent before being enrolled in the study.

**Table 1:** Eligibility criteria for the QUEST STEMI study.

**Inclusion criteria**	**Exclusion criteria**
• Patients with symptoms of myocardial infarction for >30 min	• Patients who have received fibrinolytic therapy for index STEMI event
• ST-segment elevation in ECG	• Known intolerance to heparin or anti-platelet medication
• Patients undergoing primary PCI and manual aspiration thrombectomy (at the discretion of the treating physician) within 12 h from symptom onset	
• Written informed consent	

### Angiographic Analysis, ECG, and Thrombus Aspiration Procedure

Before primary PCI, each patient received guideline-directed pharmaceutical therapy (unfractionated heparin (100 IU/kg) and a loading dose of aspirin (325 mg) and either ticagrelor (180 mg) or clopidogrel (600 mg) (12). Intravenous GP IIb/IIIa inhibitors were administered at the interventionalist's discretion. Thrombus aspiration was undertaken according to standard practices, as described (12, 22). Briefly, after crossing the lesion with a wire, the thrombectomy catheter was advanced proximal to the occluded segment. Continuous manual suction was recommended *via* a proximal-to-distal approach, so that active aspiration was initiated before the catheter crossed the thrombotic occlusion (8, 10). The thrombectomy catheter was slowly passed through the lesion multiple times (at least two), so that a minimum of 40 cc of blood and material were extracted.

A 12-lead ECG was obtained at presentation and 90 min post-intervention. ST-segment deviation was assessed, as previously described (24). Based on the degree of resolution of ST-segment elevation, patients were classified into three groups: (1) complete ST resolution (>70%); (2) partial ST resolution (30–70%); and (3) absent ST resolution (<30%).

Coronary angiograms were analyzed by two experienced interventional cardiologists (GSi, GSo). Angiographic thrombus burden was assessed based on the modified TIMI (thrombolysis in myocardial infarction) thrombus classification scale by Sianos et al. (25). According to this classification, patients with TIMI Grade 5 thrombus are classified to another thrombus category (G0–G4) post-flow achievement with either guidewire crossing or a small balloon. Furthermore, baseline, post-MATh, and post-procedural antegrade coronary flow was evaluated based on TIMI classification (26). Reference vessel diameter (RVD), minimum luminal diameter, percentage of diameter stenosis, and lesion length were also calculated using quantitative coronary angiography. Presence of distal embolization (21) and angiographically evident residual thrombus were also recorded. For the present analysis, a patient was regarded to have angiographically evident residual thrombus, if modified TIMI thrombus grades 2–4 were present (27).

### Micro-CT Analysis

**Figure 1:**
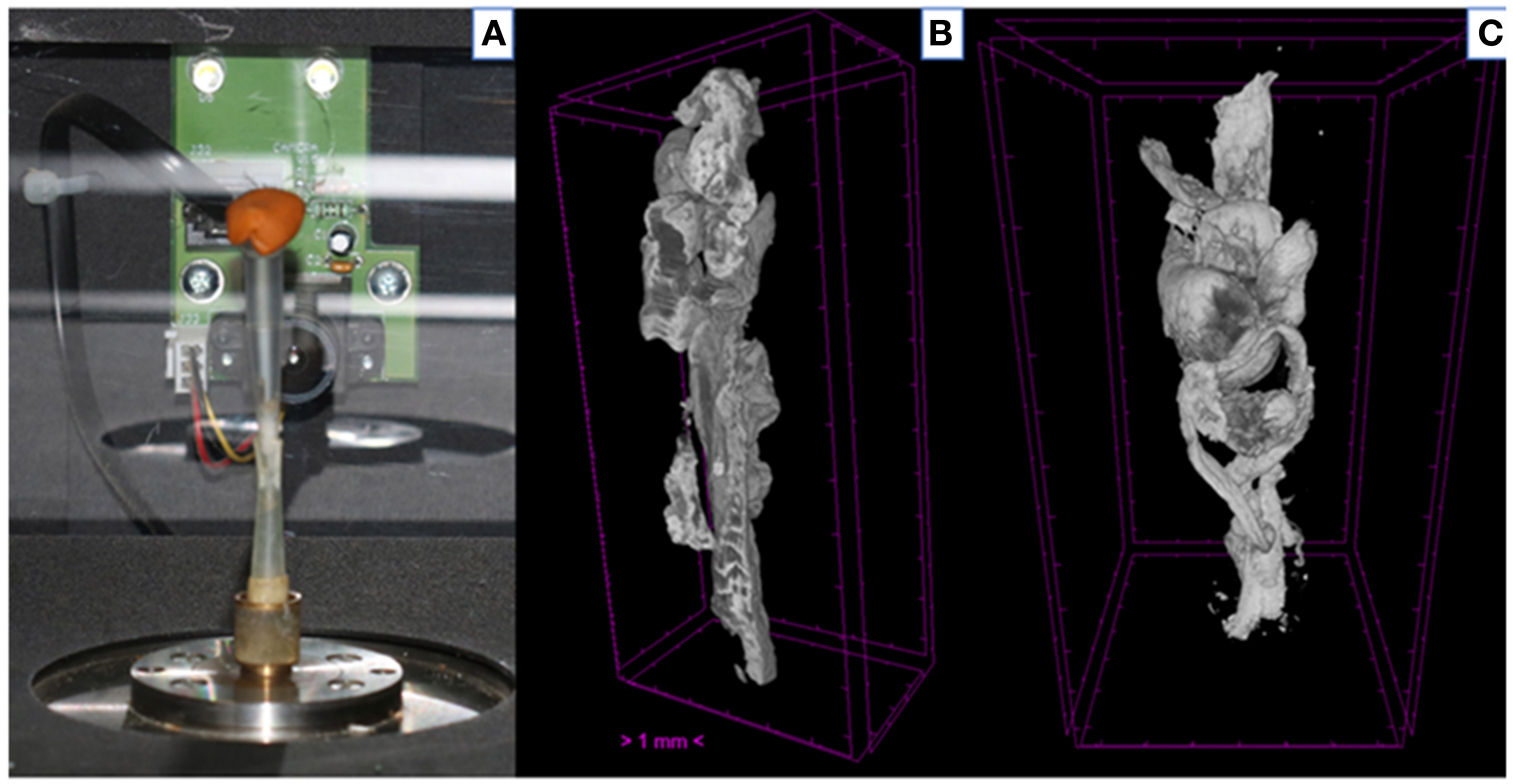
Thrombus analysis *via* micro-computed tomography. **(A)** Thrombi are mounted on a specific head inside SkyScan 1172; **(B,C)** Representative computer generated renderings of thrombi. Clots were stained using 0.3% phosphotungstic acid and scanned *via* SkyScan 1172. NRecon (Bruker, Kontich, Belgium) software was used for the reconstruction of the projections.

**Figure 2:**
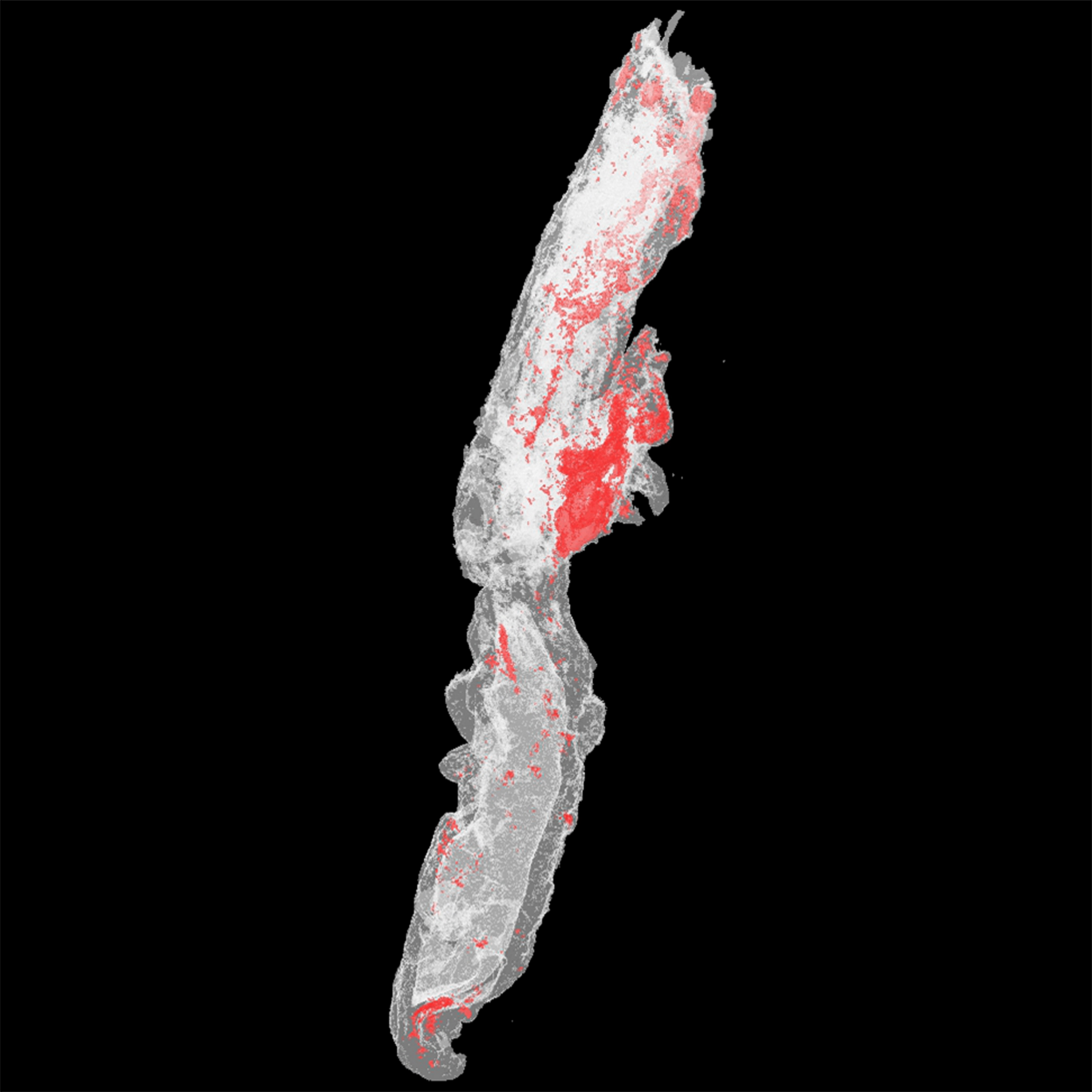
3D color visualization of a thrombus sample. A 3D model of a thrombus sample was created using CTAn software (Bruker, Kontich, Belgium), and color visualization was performed *via* CTVol software (Bruker, Kontich, Belgium). Erythrocyte-rich regions were rendered in red, whereas platelet-rich regions were rendered in white.

The detailed protocol for the micro-CT scanning and analysis of aspirated thrombi has been previously described (22). Briefly, extracted thrombotic material was initially preserved in 10% formalin for 24 h and then successively dehydrated in ethanol solutions up to 70% and stained using 0.3% phosphotungstic acid (PTA) in 70% ethanol according to Metscher's protocol (28). All scans were performed with a SkyScan 1172 micro-tomograph (Bruker, Kontich, Belgium, Figure 1A) at the Hellenic Center for Marine Research (HCMR) (48 kV, 204 μA, no filter, 360° rotation). Projection images were reconstructed into cross sections using SkyScan's NRecon software (Bruker, Kontich, Belgium). The cross-section images were loaded into the software CT Analyser v.1.14.4.1 (CTAn, Bruker, Kontich, Belgium) to extract measurements for the volume and density of thrombi (Figures 1B,C) as mean grayscale values (±Standard Deviation), which were also converted to Hounsfield units (HU). The presence of different cell types within a thrombus was also quantified by comparing their density with *in-vitro* produced thrombi with known homogeneous composition, as described (22). The range of grayscale values used for the full thrombus specimen was 25–255. Red (erythrocyte-rich) thrombi showed densities 80–255 grayscale values, whereas clots with the highest platelet content (white thrombi) had densities in the range 25–80 (Figure 2). Analysis of the thrombi was undertaken by two independent blinded assessors.

## Statistical Analysis

Statistical analysis was performed using SPSS v26 (SPSS software, Chicago, IL, USA) software. Continuous data are presented as mean and standard deviation, whereas categorical variables are expressed as counts and percentages. The inter- and intraobserver reproducibility of thrombus measurements were assessed based on data obtained from a subset of 34 subjects (30% of study population), performing Spearman's correlations, and the intraclass correlation coefficient (ICC) (29, 30).

The data were analyzed by non-parametric tests as indicated by the Shapiro–Wilk-test for normality. Group differences were tested using the Wilcoxon–Mann–Whitney-test for continuous measures. The Kruskal–Wallis *H*-test was used to compare between two or more groups of an independent variable on a continuous or ordinal dependent variable. Univariable analysis was initially carried out to clarify the association of demographic characteristics, history of smoking, medical history, statin, antiplatelet or anticoagulant use, pain-to-balloon time, pre-procedural TIMI flow, RVD, and culprit vessel with extracted thrombus characteristics (volume, surface, and density) and with angiographic and electrocardiographic outcomes. The variables with statistical significance (*p*-value <0.05) were included in the multivariable regression models.

Linear and logistic regression using stratified bootstrapping to account for the non-parametric nature of the data was used to identify independent predictors of thrombus characteristics and of angiographic outcomes. Correlation coefficients were investigated to address potential multicollinearity among the predicting variables in the created regression models (31). Multivariable logistic regression models, including both volume and surface as independent variables, demonstrated potential multicollinearity (correlation coefficients higher than 0.89). Therefore, thrombus volume and surface were not included in the same model as independent predictors. *R, R*^2^, Durbin–Watson, and Nagelkerke *R*^2^ metrics along with *p*-values are reported for the linear and logistic models, respectively.

## Results

### Study Population

During the study period, 113 consecutive patients were enrolled based on the study eligibility criteria. The baseline clinical and angiographic characteristics are shown in Tables 2, 3. Mean age was 60.05 (±12.12) years, and 89 patients (78.8%) were male. Smoking was reported by 69.9% of participants, and reperfusion was achieved on an average 330.2 (±245.74) min from symptom onset. A no-reflow phenomenon was observed in 13.3% of patients and distal embolization in 23.9% of the participants. Residual thrombus was angiographically evident in 14 patients (12.4%), whereas MBG was equal to 0 or 1 in 36 (31.86%) of patients.

**Table 2:** Demographics and baseline characteristics.

**Demographics and baseline characteristics**	**Mean (±SD) for continuous variables; count (*n*) with percentage (%) for categorical variables**
**Age**	60 (±12)
**Male** *n* (%)	89 (78.80%)
**Medical history:** ***N*** **(%)**	
A. Smoking	79 (69.90%)
B. Hypertension	37 (32.70%)
C. Dyslipidemia	23 (20.40%)
D. Diabetes mellitus	19 (16.80%)
E. Coronary artery disease	17 (15.00%)
**Pain-to-balloon time (mean, range of minutes)**	330 (30–720)
**Prior medication** ***N*** **(%)**	
I. Aspirin	14 (12.40%)
II. Clopidogrel	6 (5.30%)
III. Statins	19 (16.80%)
IV. Anti-coagulant	3 (2.70%)
V. Beta blockers	13 (11.50%)

**Table 3:** Percutaneous coronary intervention procedure details and angiographic outcomes.

**Infarct-related artery**	
LM	2 (1.80%)
LAD	47 (42.34%)
LCx	17 (15.32%)
RCA	45 (40.54%)
**Pre-procedural TIMI flow**	
0	69 (62.16%)
1	20 (18.01%)
2	10 (9.00%)
3	12 (10.81%)
Modified TIMI thrombus grade classification^*^	
0	0
1	0
2	5 (4.50%)
3	31 (27.93%)
4	61 (54.95%)
**Use of stenting—no. (%)**	103 (91.20%)
**GP2B3A antagonist use**	41(36.30%)
**Post-procedural results**	
**Final TIMI flow**	
0	2 (1.80%)
1	1 (0.90%)
2	18 (16.22%)
3	90 (81.08%)
**Angiographic no reflow**	15 (13.30%)
**Distal embolization**	27 (23.90%)
**Angiographically evident residual thrombus burden**	14 (12.40%)
Myocardial blush grade^*^	
0	31 (27.43%)
1	5 (4.4%)
2	15 (13.27%)
3	47 (41.59%)
**ECG: ST-segment resolution**	
Complete (>70%)	59 (52.21%)
Partial (30–70%)	41 (36.28%)
Absent (<30%)	13 (11.50%)
**In-hospital death**	10 (8.85%)

* *In 14 patients (12.61%), thrombus grade could not be classified according to modified TIMI thrombus grade classification (25), and in 15 patients (13.27%) myocardial blush grade could not be calculated, because of inadequate angiographic documentation*.

*PCI, percutaneous coronary intervention; LM, left main artery; LAD, left anterior descending; LCx, left circumflex; RCA, right coronary artery; IRA, infarct-related artery; TIMI, thrombolysis in myocardial infarction; ECG, electrocardiography, GP2B3A, glycoprotein IIB/IIIA*.

### Micro-CT Findings on Extracted Thrombus Burden

Micro-tomography effectively quantified the volume, surface, and density of all aspirated thrombi. No sample disintegration was observed, and hence all thrombi were suitable for micro-CT scanning. The mean extracted thrombus volume, surface, and density were 15.71 (± 20.10) mm^3^, 302.89 (± 692.54) mm^2^, and 3139.04 (± 901.88) HU, respectively.

Intraobserver and interobserver reliabilities were high for all thrombus volume (interobserver: 0.995; intraobserver: 1.000), thrombus surface (interobserver: 0.999; intraobserver: 0.999), and thrombus density (interobserver: 0.982; intraobserver: 0.982). Interclass correlation coefficients for thrombus volume, surface, and density were equal to 0.995 (95% C.I.: 0.981–0.998), 0.995 (95% C.I.: 0.991–0.996), and 0.987 (95% C.I.: 0.966–0.993), respectively.

### Association of Extracted Thrombus Burden Characteristics With Intracoronary Thrombus Classification

**Figure 3:**
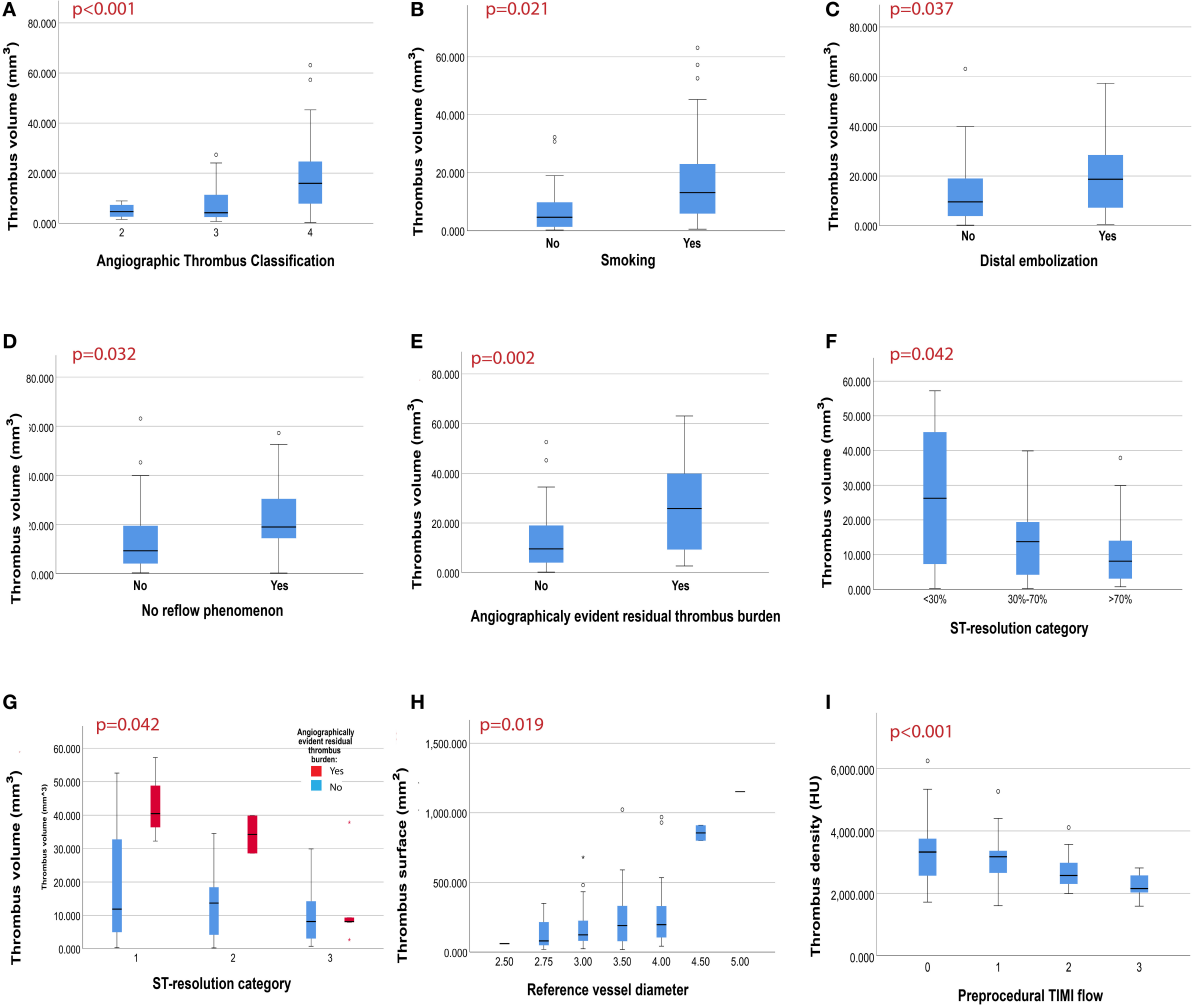
Main findings of the QUEST STEMI study. **(A)** Association of thrombus volume with angiographic thrombus classification by modified TIMI thrombus grade classification [Grade 2: 4.64 (2.66–8.92) mm^3^, Grade 3: 4.21 (3.72–8.80) mm^3^, Grade 4: 15.94 (10.60–19.51) mm^3^]. **(B)** Association of thrombus volume with smoking history [yes: 13.14 (9.31–16.35) mm^3^, no: 4.64 (2.66–8.69) mm^3^]. **(C–E)** Association of thrombus volume with angiographic outcomes {**(C)** distal embolization [yes: 18.70 (9.27–24.52) mm^3^, no: 9.61 (6.61–13.82) mm^3^], **(D)** no-reflow phenomenon: [yes: 18.98 (15.79–32.26) mm^3^, no: 9.31 (6.61–13.65) mm^3^], and **(E)** angiographically evident residual thrombus [yes: 25.81 (9.31–39.87) mm^3^, no: 9.61 (6.61–13.65) mm^3^]}. **(F,G)** Association of thrombus volume with electrocardiographic outcomes [complete ST resolution; 8.12 (5.93–10.31) mm^3^, partial ST resolution; 13.74 (6.42–16.97) mm^3^ and absent ST resolution: 26.26 (7.31–45.26) mm^3^]. **(H)** Association of thrombus surface with reference vessel diameter [RVD: 2.5 mm: 150.30 (±175.22) mm^2^, 3 mm: 187.54 (±152.22) mm^2^, 3.5 mm: 233.21 (±198.23) mm^2^, 4 mm: 539.17 (±1388.82) mm^2^, 4.5 mm: 855.73 (±78.26) mm^2^]. **(I)** Association of thrombus density with pre-procedural TIMI flow [TIMI 0: 3322 (3023–53523) HU, TIMI I: 3171 (2863–3331) HU, TIMI II: 2574 (2307–3564) HU, TIMI III: 2152 (2019–2594) HU].

Aspirated thrombus volume and surface were significantly higher (*p*-value <0.001) in patients with higher intracoronary angiographic thrombus burden according to modified TIMI thrombus grade classification (Figure 3A). Similarly, aspirated thrombus density analysis revealed that higher values of density (indicating a higher proportion of erythrocytes within the clot) were significantly correlated with larger intracoronary angiographic thrombus burden according to modified TIMI thrombus grade classification (*p*-value = 0.037).

### Determinants of Extracted Thrombus Burden Characteristics

Univariable analysis revealed that male gender, history of smoking, RVD, and right coronary artery (RCA) as the culprit vessel are significantly associated with higher extracted thrombus volume (*p*-values = 0.044, 0.001, 0.021, 0.017, respectively). Preprocedural TIMI flow was not associated with aspirated volume (*p*-value = 0.794). On bootstrapped multivariable linear regression analysis (Table 4), RVD (*p*-value = 0.011), RCA as the culprit vessel (*p*-value = 0.039), and history of smoking (*p*-value = 0.027) were independent predictors of higher extracted thrombus volume (Figure 3B).

**Table 4:** Determinants of extracted thrombus burden characteristics.

**Determinants of TB**	**Volume**		**Surface**	
	**Univariable *p*-value and stand. beta**	**Multivariable *p*-value and stand. beta**	**Univariable *p*-value and stand. beta**	**Multivariable *p*-value and stand. beta**
**Gender**	*p* = 0.044	*p* = 0.419	*p* = 0.081	*p* = 0.729
	*B* = −0.171	*B* = −0.034	*B* = −0.112	*B* = −0.026
**History of smoking**	*p* = 0.001	***p*** **=** **0.027**	*p* = 0.003	*p* = 0.193
	*B* = 0.285	***B*** **=** **0.193**	*B* = 0.226	*B* = 0.102
**RVD**	*p* = 0.021	***p*** **=** **0.011**	*p* = 0.014	***p*** **=** **0.018**
	*B* = 0.294	***B*** **=** **0.272**	*B* = 0.236	***B*** **=** **0.348**
**RCA as the culprit vessel**	*p* = 0.017	***p*** **=** **0.039**	*p* = 0.012	***p*** **=** **0.019**
	*B* = 0.039	***B*** **=** **0.203**	*B* = −0.040	***B*** **=** **0.219**
Model metrics:		Durbin–Watson = 1.240, *R* = 0.444, adjusted *R*^2^ = 0.164, *p* < 0.001		Durbin–Watson = 1.320, *R* = 0.495, adjusted *R*^2^ = 0.205, *p* < 0.001

*RCA refers to right coronary artery and RVD refers to reference vessel diameter*.

*Bold p- and beta- values represent statistically significant outcomes of multivariable analysis*.

Higher extracted thrombus surface was—both univariably and multivariably (Table 4)—significantly linked with larger RVD (*p*-value = 0.018) and RCA as the culprit vessel (*p*-value = 0.019; Figure 3H), whereas the association of surface with preprocedural TIMI flow was not significant (*p*-value = 0.860).

It is worth mentioning that the Durbin–Watson values of our regression models (for volume: 1.240 and for surface: 1.320) indicate that there is no significant autocorrelation. Despite, *R*^2^-values of these models propose a significant, but weak, predictive value (*R*^2^ < 0.300).

As for thrombus density, univariable analysis demonstrated that higher density values (erythrocyte-rich clots) were significantly correlated with worse pre-procedural TIMI flow (*p*-value < 0.001; Figure 3I), but no other statistically significant predictor of density was revealed and hence no multivariable regression model was performed.

### Association of Extracted Thrombus Burden Characteristics With Angiographic Outcomes

Higher extracted thrombus volume was significantly linked with angiographic outcomes suggestive of poor patient prognosis, including distal embolization (*p*-value = 0.037), and no reflow phenomenon (*p*-value = 0.032; Figures 3C,D). Additionally, angiographic evidence of residual thrombus was more frequent in patients with larger aspirated thrombus (*p*-value = 0.002; Figure 3E). Furthermore, a non-significant trend toward worse MBG in patients with larger extracted thrombus volume was observed (*p* = 0.073), hence multivariable regression analysis on MBG was not performed.

Bootstrapped multivariable logistic regression analyses (Table 5) showed that aspirated thrombus volume remained an independent predictor for (i) distal embolization (*p*-value = 0.007), (ii) no-reflow phenomenon (*p*-value = 0.002) together with smoking (*p* = 0.015), and (iii) angiographically evident residual thrombus (*p*-value = 0.007).

**Table 5:** Association of extracted thrombus volume with angiographic outcomes.

Multivariable analysis^*^	**Angiographic outcomes**	**1. Distal embolization**		**2. No-reflow**		**3. Angiographically evident residual thrombus**	
	**Variables**	**Standardized beta**	***p*-value**	**Standardized beta**	***p*-value**	**Standardized beta**	***p*-value**
	**Volume**	0.060	**0.007**	0.085	**0.002**	0.078	**0.007**
	**Age**	0.047	**0.039**	−0.025	0.421	−0.086	0.104
	**Smoking**	−0.125	0.866	1.75	**0.015**	0.499	0.540
	**RCA as the culprit vessel**	0.706	0.337	−0.522	0.345	−2.790	**0.025**
Model metrics:		χ^2^ = 13.526, Nagelkerke *R*^2^ = 0.182, *p* = 0.035		χ^2^ = 17.862, Nagelkerke *R*^2^ = 0.282, *p* = 0.013		χ^2^ = 38.488, Nagelkerke *R*^2^ = 0.587, *p* < 0.001	

* *Each column represents a different multivariable regression model for each dependent predictor (1: distal embolization, 2: no reflow phenomenon, and 3: angiographically evident residual thrombus). Each row represents a different independent predictor. Statistically significant results are marked in bold. RCA refers to right coronary artery*.

Similarly, bootstrapped multivariable logistic regression analysis on thrombus surface (Table 6) showed that the higher surface of aspirated thrombus was independently associated with (i) distal embolization (*p*-value = 0.028) along with age (*p*-value = 0.024), (ii) no-reflow phenomenon (*p*-value = 0.006) along with smoking (*p*-value = 0.021), and (iii) angiographically evident residual thrombus (*p*-value = 0.002) along with RCA (*p*-value = 0.016). On the other hand, multivariable regression analysis on MBG was not performed, since univariable analysis did not yield statistically significant results (*p*-value = 0.226).

**Table 6:** Association of extracted thrombus surface with angiographic outcomes.

Multivariable analysis^*^	**Angiographic outcomes**	**1. Distal embolization**		**2. No-reflow**		**3. Angiographically evident residual thrombus**	
	**Variables**	**Standardized beta**	***p*-value**	**Standardized beta**	***p*-value**	**Standardized beta**	***p*-value**
	**Surface**	0.002	**0.028**	0.003	**0.006**	0.005	**0.002**
	**Age**	0.051	**0.024**	−0.015	0.566	−0.059	0.121
	**Smoking**	−0.312	0.611	1.595	**0.021**	0.286	0.776
	**RCA as the culprit vessel**	0.222	0.672	−0.526	0.421	−2.824	**0.016**
Model metrics:		χ^2^ = 13.908, Nagelkerke *R*^2^ = 0.196, *p* = 0.042		χ^2^ = 13.025, Nagelkerke *R*^2^ = 0.211, *p* = 0.011		χ^2^ =35.119, Nagelkerke *R* ^2^ = 0.544, *p* < 0.001	

* *Each column represents a different multivariable regression model for each dependent predictor (1: distal embolization, 2: no reflow phenomenon, and 3: angiographically evident residual thrombus). Each row represents a different independent predictor. Statistically significant results are marked in bold. RCA refers to right coronary artery*.

Of note, multivariable model metrics indicate that the predictive models for angiographically evident residual thrombus explain the most of the variation compared with the other models (volume, smoking, age, RCA: Nagelkerke *R*^2^ = 0.587 and surface, age, smoking, RCA: Nagelkerke *R*^2^ = 0.544). However, the other multivariable models created for the prediction of distal embolization and no-reflow phenomenon have weak, but statistically significant, predictive value (Nagelkerke *R*^2^ < 0.300).

Additionally, thrombus density was not associated with distal embolization (*p*-value = 0.246), no-reflow phenomenon (*p*-value = 0.859), angiographically evident residual thrombus (*p*-value = 0.549), or MBG (*p*-value = 0.155) in univariable analysis and therefore no multivariable regression model was executed.

### Association of Extracted Thrombus Burden Characteristics With Electrocardiographic Outcomes

ECG analysis revealed that patients with higher aspirated thrombus volume and surface developed significantly less ST resolution (*p*-value = 0.042 and 0.023, respectively). After classifying patients by the presence of angiographically evident residual thrombus, ST-segment resolution was less in patients with angiographically evident residual thrombus and particularly in those with higher aspirated thrombus volume (Figures 3F,G). On the other hand, no significant correlation between thrombus density and ST-segment resolution (*p*-value = 0.451) was observed.

## Discussion

**Figure 4:**
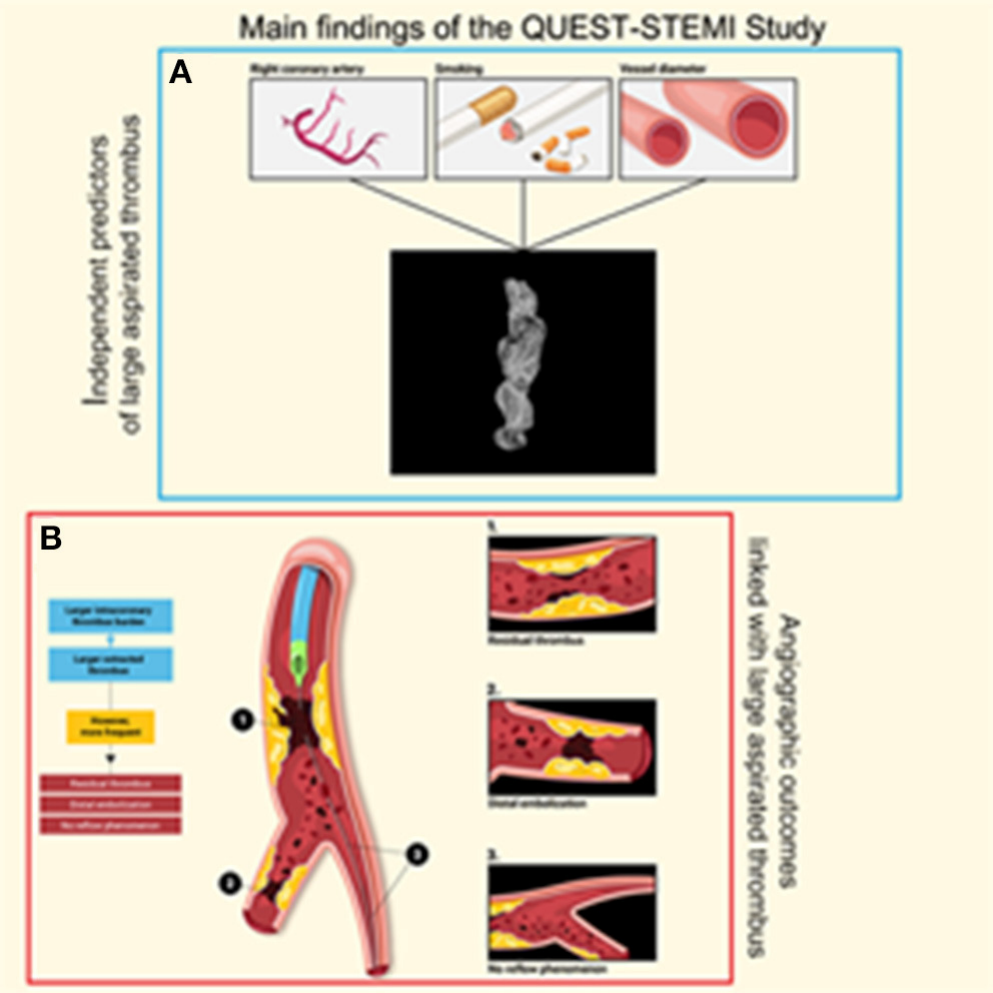
Visual overview of the main findings of the QUEST STEMI study. **(A)** Independent predictors of large aspirated thrombus and **(B)** Angiographic outcomes linked with large aspirated thrombus.

To our knowledge, this is the first study to comprehensively evaluate extracted thrombotic material in patients with STEMI using micro-CT. The main findings of our study (Figure 4) were as follows: (1) thrombus analysis by micro-CT is feasible, reliable, and reproducible; (2) larger thrombus (higher volume and surface) was extracted in patients with high intracoronary TB under angiographic imaging; (3) higher aspirated thrombus volume and surface were associated with adverse angiographic outcomes, including distal embolization and no-reflow phenomenon; (4) angiographically evident residual thrombus was more frequent among patients with larger retrieved thrombus; (5) a non-significant trend (*p* = 0.073) toward worse myocardial blush grade in patients with larger extracted thrombus volume was observed; (6) aspirated thrombus volume and surface were significantly higher in smokers; and (7) worse pre-procedural TIMI flow was observed in thrombi with higher density.

Thrombus is a typical histopathologic characteristic of patients suffering from STEMI, which has been linked with worse patient prognosis (32). Qualitative and quantitative assessment of coronary thrombi has been a challenge. Recently, OCT has been employed to effectively quantify and characterize intracoronary thrombus before (15) and after (14, 16) MATh.

In this study, using a novel technology (micro-CT) we aimed to develop a methodology for the visualization and the quantitative 3D analysis of extracted thrombotic material in patients with STEMI undergoing MATh during pPCI. Micro-CT is an established imaging modality, which facilitates high-resolution, non-destructive visualization of 3D structures along with quantitative volumetric measurements and tissue characterizations. Our results indicate that this method shows high reliability and repeatability for measuring the volume, surface, and density of extracted clots. Interestingly, micro-CT-quantified thrombus burden was strongly correlated with the intracoronary TB under angiographic imaging according to modified TIMI thrombus grade classification (25), suggesting that higher amount of thrombus is retrieved in patients with larger thrombotic load as seen in angiography.

In cases of large TB, fully optimizing stent expansion, while simultaneously preventing distal embolization and protecting the distal coronary vasculature, remains a clinical challenge, given the fact that routine MATh has not been proven effective (12). The inability of MATh to demonstrate a clinical benefit (8, 33, 34) might be the result of current MATh technology limitations, including inadequate retrieval of thrombus, thrombus embolization downstream prior to aspiration, and limited ability to deal with and not to dislodge large organized thrombotic material to other vascular territories during removal of the aspiration catheter (13). Interestingly, several trials using OCT demonstrated large residual thrombus after aspiration with current thrombus aspiration devices (16, 35). Hence, effective thrombus removal is crucial.

Our study is the first to show that angiographic outcomes, linked with worse prognosis, including distal embolization (5), no-reflow phenomenon (36), and angiographically evident residual thrombus, are more frequent among patients with larger retrieved thrombus, as assessed quantitatively. Similarly, we observed a trend toward worse MBG in patients with larger aspirated thrombus volume. Moreover, in these patients a lower extent of ST-segment resolution was observed, which could be potentially attributed to a higher prevalence of residual thrombus. These findings suggest that, despite retrieving higher thrombus load, current MATh technology fails to adequately deal with thrombotic material in patients with large TB, who are at higher risk for adverse outcomes and who would theoretically benefit most from effective thrombus removal. Therefore, given the fact that residual thrombus due to ineffective thrombectomy has been associated with impaired microcirculatory perfusion and more significant myocardial injury (14), further studies are warranted to explore novel more effective thrombus aspiration technologies with the potential to improve patient outcomes (37).

Regarding variables correlated with larger extracted thrombus, we observed that aspirated thrombus volume was higher among smokers. This finding is in line with previous studies showing a greater thrombus burden in smokers (38). Smoking may induce a hypercoagulable state increasing blood viscosity (39) and promoting platelet aggregation and thrombogenesis. These mechanisms could also explain our finding that smoking constitutes an independent risk factor for no-reflow phenomenon, although this relationship has not been yet confirmed (40). Apart from smoking, our data suggest that lumen diameter and RCA are predictive factors for larger retrieved thrombi. Larger coronary vessels can accommodate greater amounts of thrombotic material. Another potential explanation could be lower shear stress observed in vessels with greater diameter, as shear stress has been shown to influence the mechanisms supporting platelet aggregation and adhesion to the thrombogenic area through different pathways (41). Current literature also supports that thrombi in RCA tend to be larger possibly due to proximal propagation of thrombotic material related to fewer branch points (42, 43).

Moreover, micro-CT analysis of thrombus density demonstrated that patients with clots of high density (erythrocyte-rich thrombi) experienced worse pre-procedural TIMI flow. This finding is in line with previous histopathologic reports (44) showing that TIMI flow was worse in patients with red clots, compared to patients with white clots. A similar OCT-based study by Higuma et al. (35) showed that thrombi with greater erythrocyte-positive area were associated with worse angiographic outcomes.

## Limitations

Our study has several limitations. Firstly, all patients were enrolled from a single center, which could limit the generalizability of our results. Secondly, the study lacks intracoronary imaging data, thus the presence of residual thrombus was based on visual evaluation. Moreover, MATh was performed at the discretion of the interventionalist executing the pPCI and therefore the possibility of selection bias cannot be excluded. It is also possible that periprocedural IIBIIIA inhibitor administration and operators' technique could have affected the outcomes of our study. However, all operators were strongly encouraged to follow the same thrombectomy procedure. Thus, derived outcomes should not have been substantially affected, by inconsistent aspiration techniques. Additionally, formalin used to preserve extracted clots may have caused thrombus shrinkage and subsequent underestimation of thrombus measurements. Similarly, sample staining with PTA has altered the measured density of the thrombotic material. However, both formalin fixation and PTA staining affected equally all specimens, since the same process was applied to all of them. Hence, we expect that these processes did not influence the comparability of our measurements. Last, this trial was not designed to investigate potential associations between micro-CT findings and hard clinical outcomes.

In conclusion, novel imaging techniques, such as micro-CT, can be employed for accurate and reproducible assessment of extracted thrombotic material, paving the way for more extensive research in this field. Our results indicate that the inadequacy of MATh to provide the anticipated benefit to patients with STEMI and large thrombotic load could be attributed to the limitations of current aspiration thrombectomy devices. A major question to be addressed by future studies is whether the development and optimization of more efficient MATh devices could facilitate effective thrombus removal and minimal residual thrombus burden and subsequently improve short- and long-term outcomes of coronary procedures in this challenging setting.

## Data Availability Statement

The datasets presented in this article are not readily available because data are available from corresponding author upon reasonable request and with permission of AHEPA University Hospital. Requests to access the datasets should be directed to Georgios Sianos, e-mail: gsianos@auth.gr.

## Ethics Statement

The studies involving human participants were reviewed and approved by Scientific Committee, University General Hospital of Thessaloniki, AHEPA and Medical Ethics Committee, Medical School, Aristotle University of Thessaloniki. The patients/participants provided their written informed consent to participate in this study.

## Author Contributions

GSi, HK, JM, and GG developed the concept and the trial protocol. EK, AP, and GSi wrote the manuscript. AK, GSo, and SH were responsible for coronary angiography execution. EP, KKo, and LS contributed to the research data management and statistical considerations, whereas DM, NS, and TZ were responsible for patient recruitment and follow-up execution. EC, KKe, and CA were responsible for micro-CT imaging execution, while FG contributed to the study with artificial clots creation. The principal investigator and supervisor of the whole study was GSi. All authors have read and approved the final manuscript.

## Conflict of Interest

The authors declare that the research was conducted in the absence of any commercial or financial relationships that could be construed as a potential conflict of interest.

## Abbreviations

ECG: Electrocardiogram
MACCE: Major Adverse Cardiac and Cerebrovascular events
MATh: Manual Aspiration Thrombectomy
MBG: Myocardial Blush Grade
Micro-CT: Micro-Computed Tomography
OCT: Optical Coherence Tomography
PCI: Percutaneous Coronary Intervention
RVD: Reference Vessel Diameter
STEMI: ST-Elevated Myocardial Infarction
TIMI: Thrombolysis In Myocardial Infarction
TB: Thrombus Burden.
